# Low‐Dose Aspirin Induced Gastric Ulcer in a Patient With TIA: A Clinical Caution for Aspirin Therapy Without Gastroprotection

**DOI:** 10.1002/ccr3.71913

**Published:** 2026-01-21

**Authors:** Kainat Husain, Muhammad Ibrahim, Fazeela Bibi, Khalil El Abdi, Bilal Aslam, Abdullah Saud, Vohra Maham Hassan, Aminullah Betanai, Muhammad Muneeb, Said Hamid Sadat

**Affiliations:** ^1^ Jawaharlal Nehru Medical College Aligarh India; ^2^ Bannu Medical College Bannu Pakistan; ^3^ Jinnah Medical and Dental College Karachi Pakistan; ^4^ Faculty of Medicine and Pharmacy of Rabat Mohammed V University Rabat Morocco; ^5^ University of Lahore Lahore Pakistan; ^6^ Gajju Khan Medical College Swabi Pakistan; ^7^ Holy Family Hospital Rawalpindi Rawalpindi Pakistan; ^8^ Khyber Medical College Peshawar Pakistan; ^9^ Kabul University of Medical Sciences Abu Ali Ibn Sina Kabul Afghanistan

**Keywords:** gastroprotection, low‐dose aspirin, proton pump inhibitor, secondary stroke prevention, transient ischemic attack, upper gastrointestinal bleeding

## Abstract

Balancing the cerebrovascular benefits of low‐dose aspirin (LDA) with its significant gastrointestinal (GI) bleeding risk is a critical challenge in secondary stroke prevention. We describe a cautionary case of a 60‐year‐old male who, 6 months after starting LDA for a transient ischemic attack (TIA), presented with a life‐threatening upper GI bleed. The patient had not been prescribed a prophylactic proton pump inhibitor (PPI). Investigations revealed a hemoglobin of 9.6 g/dL and multiple gastric ulcers on endoscopy, with negative testing for *Helicobacter pylori* . This case of a preventable, severe adverse event serves as a stark reminder that guideline‐recommended gastroprotection is not optional but essential. Systematic risk assessment and PPI co‐prescription in at‐risk individuals are imperative for the safe and effective use of long‐term antiplatelet therapy.

## Key Clinical Message

Systematic gastrointestinal risk assessment is mandatory before initiating long‐term aspirin. For at‐risk patients, co‐prescribing a proton pump inhibitor is an essential safety measure that makes guideline‐based prophylaxis a core component of safe and effective secondary stroke prevention.

## 1 Introduction

Stroke is a leading cause of mortality and long‐term disability worldwide [1], with ischemic events accounting for the majority of cases [2]. A transient ischemic attack (TIA) is a critical warning sign, as it is a powerful predictor of a major stroke, often within the subsequent 48 h [3]. Consequently, the prompt initiation of secondary prevention is paramount [3]. Antiplatelet therapy, particularly with low‐dose aspirin (LDA), remains the cornerstone of management for patients with non‐cardioembolic ischemic stroke or TIA [4]. Its efficacy is well‐established; large‐scale analyses have shown that LDA reduces the risk of early recurrent ischemic stroke by approximately 60% and debilitating or fatal ischemic stroke by nearly 70% [5, 6].

Despite its undisputed benefits for cerebrovascular protection, LDA therapy carries a significant and inherent risk of serious adverse effects, primarily involving the upper gastrointestinal (GI) tract [7]. Aspirin irreversibly inhibits cyclooxygenase‐1 (COX‐1), leading to disruption in the synthesis of protective prostaglandins which maintain gastric mucosal integrity and mucosal defense mechanisms [8]. This mechanism substantially increases the risk of peptic ulcer disease (PUD), although symptomatic ulcer incidence in the general population is generally lower than previously suggested, with annual rates closer to 1 per 1000 persons but higher in selected high‐risk groups [9]. Clinical consequences of aspirin‐associated GI injury range from dyspepsia to life‐threatening complications including hematemesis, melena due to GI bleeding, and perforation [7]. These risks are markedly elevated in older adults and individuals with a prior history of peptic ulcers or *Helicobacter pylori* infection, for whom eradication therapy may reduce ulcer complications [10].

To mitigate this risk, major clinical practice guidelines strongly recommend a proactive approach to gastroprotection for patients on long‐term LDA who are at high risk for GI complications [11]. The primary strategies include screening for and eradicating *H. pylori* infection and, crucially, the co‐prescription of a proton pump inhibitor (PPI) [12]. PPI co‐therapy has been shown to significantly reduce the risk of upper GI bleeding without compromising the cardiovascular efficacy of aspirin [13]. However, a gap often exists between these evidence‐based recommendations and clinical practice. We present a cautionary case of a 60‐year‐old male who developed a severe bleeding gastric ulcer 6 months after starting LDA for a TIA, highlighting the critical consequences of omitting guideline‐recommended gastroprotection.

## 2 Case Description

A 60‐year‐old male presented to the emergency department with a three‐day history of melena and a two‐day history of hematemesis. He described one daily episode of black, tarry stool that was difficult to flush and a single episode of projectile, coffee‐ground hematemesis 2 days prior to presentation. He denied any associated abdominal pain, dyspepsia, dysphagia, or weight loss. His past medical history was significant for a TIA 6 months prior, which had manifested as self‐resolving slurred speech and dizziness. His medical history also included chronic hypertension. Following the TIA, he was prescribed a secondary prevention regimen of aspirin (75 mg daily), a combination of amlodipine/valsartan (5/80 mg daily), and atorvastatin (10 mg daily). Notably, no gastroprotective agent, such as a PPI, was co‐prescribed. The patient had no history of alcohol abuse, smoking, or a family history of malignancy. On physical examination, the patient appeared pale but was afebrile and hemodynamically stable, with a blood pressure of 110/70 mmHg and a heart rate of 104 beats per min. His respiratory rate was 20 breaths per min with an oxygen saturation of 98% on room air. He was alert and oriented, with a Glasgow Coma Scale (GCS) score of 15/15. The abdominal examination was unremarkable, with no tenderness or organomegaly. A digital rectal examination confirmed the presence of melena.

## 3 Investigations and Differential Diagnosis

**FIGURE 1:**
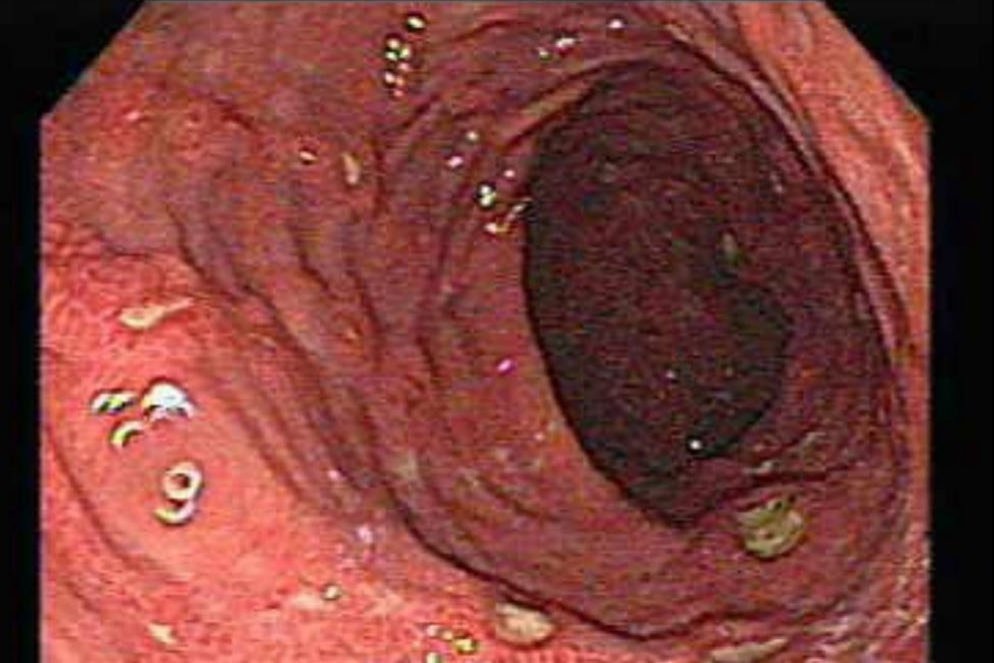
Aspirin‐induced gastric ulcers with stigmata of recent hemorrhage. Upper endoscopic view of the gastric antrum reveals multiple shallow ulcers with adherent dark clots. These findings are characteristic of recent hemorrhage (Forrest class IIb) and were attributed to low‐dose aspirin therapy in the absence of *H. pylori* .

Initial laboratory investigations revealed a normocytic, hypochromic anemia with a hemoglobin (Hb) of 9.6 g/dL (normal: 14–18 g/dL), a mean corpuscular volume (MCV) of 78 fL (normal: 77–91 fL), and a mean corpuscular hemoglobin (MCH) of 23 pg (normal: 25–32 pg). The coagulation profile was mildly deranged with an activated partial thromboplastin time (aPTT) of 38 s (normal: 25–35 s). The blood urea nitrogen (BUN) to creatinine ratio was elevated at 24:1, suggestive of an upper GI bleed. Liver function tests were within normal limits. To rule out other common causes of PUD, urea breath and stool antigen tests for *Helicobacter pylori* were performed; both were negative. Given the negative *H. pylori* testing and the patient's medication history, the bleeding peptic ulcers were attributed to LDA therapy. An urgent upper endoscopy was performed, which revealed multiple, non‐bleeding gastric ulcers in the antrum with visible clots, consistent with recent hemorrhage (Forrest class IIb) (Figure 1). Hemostasis was successfully achieved with a combination of epinephrine injection and thermocoagulation. Based on his age, shock (tachycardia), and high‐risk endoscopic stigmata, his post‐endoscopy Rockall Risk Score was 6, indicating a high risk of rebleeding (32.9%) and mortality (17.3%).

## 4 Treatment, Outcome, and Follow‐Up

The patient was admitted to a high‐dependency unit for close monitoring. Aspirin was immediately discontinued. He was initiated on intravenous fluids, tranexamic acid, and a continuous infusion of omeprazole (80 mg bolus followed by 8 mg/h) for 72 h, consistent with guidelines for high‐risk bleeding ulcers. His condition stabilized rapidly following endoscopic intervention, with no further episodes of hematemesis or melena during his 2‐day hospital stay. He was transitioned to high‐dose oral omeprazole (40 mg twice daily). At discharge, his hemoglobin was stable. In accordance with current evidence demonstrating superior outcomes and lower mortality, the patient was discharged on oral omeprazole (40 mg once daily) and instructed to resume aspirin (75 mg daily) 5 days post‐discharge to maintain essential secondary stroke prevention. At his 15‐day follow‐up, the patient reported complete symptomatic resolution and excellent medication adherence. A repeat complete blood count confirmed hematological recovery, with a hemoglobin of 12.01 g/dL. A follow‐up endoscopy at 12 weeks confirmed the complete healing of the gastric ulcers, with well‐formed white scars (ulcus cicatrix) at the prior sites and no evidence of residual inflammation or erosions. The definitive long‐term management plan consists of lifelong aspirin (75 mg daily) co‐prescribed with lifelong omeprazole (40 mg daily). The patient was counseled extensively on the importance of adherence and educated to avoid all over‐the‐counter non‐steroidal anti‐inflammatory drugs (NSAIDs).

## 5 Discussion

This case report details a severe, yet preventable, upper GI bleed in a patient on LDA for secondary stroke prevention. The principal finding is the direct clinical consequence of omitting guideline‐recommended gastroprotection in a patient with a clear risk factor. This case underscores the critical importance of systematic risk assessment and adherence to evidence‐based prophylactic strategies when initiating long‐term antiplatelet therapy.

The value of this report lies not in its rarity, but in its stark illustration of the persistent gap between evidence and practice. While the risks of aspirin are well‐known and clinical guidelines for their mitigation are clear, this case demonstrates a common failure point in the healthcare system: the prescription of a long‐term, high‐risk medication without the simultaneous initiation of necessary protective co‐therapy. The patient's presentation with a life‐threatening bleed was a direct result of this oversight, transforming a vital preventative therapy into a source of significant morbidity. This report serves as a practical, cautionary vignette for all clinicians involved in prescribing LDA.

The patient's risk profile merited proactive gastroprotection. At 60 years of age and on long‐term LDA, he met the criteria for increased risk where prophylactic therapy should be strongly considered, as recommended by multiple gastroenterology and cardiology societies [14]. The negative *Helicobacter pylori* testing in our patient is a key finding, as it effectively rules out the most common cause of PUD and strengthens the attribution of the gastric ulcers to aspirin's systemic inhibition of COX‐1 and subsequent disruption of mucosal defenses. This highlights that while *H. pylori* eradication is a crucial component of risk reduction, it is not a substitute for acid suppression with a PPI in at‐risk individuals continuing aspirin therapy [15].

The acute management, involving endoscopic hemostasis and initiation of high‐dose intravenous PPI, aligned with standard‐of‐care for bleeding ulcers [16]. The subsequent decision to resume aspirin 5 days post‐discharge is a critical teaching point and reflects a careful balancing of risks. Evidence from randomized trials demonstrates that discontinuing aspirin after a GI bleed significantly increases the risk of mortality from cardiovascular and cerebrovascular events, a risk that outweighs that of re‐bleeding when a PPI is used concurrently [17]. While switching to clopidogrel is a potential alternative, the combination of aspirin and a PPI has been shown to be superior to clopidogrel monotherapy in preventing recurrent ulcer bleeding in high‐risk patients [18]. This evidence provides a strong rationale for the “re‐challenge with cover” strategy employed in this case.

This case yields several actionable clinical implications. First, a formal GI risk assessment should be an integrated, routine step before initiating long‐term LDA. This includes evaluating age, history of PUD, and concomitant medication use. Second, for patients with one or more major risk factors, PPI co‐prescription should be the default practice. While concerns about potential long‐term PPI side effects (e.g., micronutrient deficiencies, infection risk) exist, the immediate and high mortality risk associated with a major GI bleed in a patient on antiplatelet therapy makes the risk–benefit analysis strongly favor gastroprotection.

From a health‐economic standpoint, this approach is highly favorable. The cost of managing a single episode of major GI bleeding—including emergency services, hospitalization, blood transfusions, and endoscopic procedures—is substantial, often exceeding tens of thousands of dollars [19]. In contrast, the daily cost of a generic PPI is minimal. Economic modeling studies have consistently shown that for patients at moderate to high risk of GI complications, the routine co‐prescription of a PPI with LDA is a dominant cost‐effective strategy that both reduces morbidity and lowers overall healthcare expenditure [20].

## 6 Conclusion

While LDA is indispensable for the secondary prevention of ischemic stroke, its prescription must be inseparable from a diligent and systematic assessment of GI bleeding risk. This case demonstrates that failure to co‐prescribe a PPI in at‐risk individuals can lead to severe, costly, and preventable harm. Integrating gastroprotective strategies into routine prescribing practice is essential to safely realize the full life‐saving potential of antiplatelet therapy.

## Author Contributions

**Kainat Husain:** conceptualization, data curation, resources, writing – original draft, writing – review and editing. **Muhammad Ibrahim:** formal analysis, project administration, validation, visualization. **Fazeela Bibi:** conceptualization, data curation, resources, writing – original draft, writing – review and editing. **Khalil El Abdi:** conceptualization, data curation, resources, writing – original draft, writing – review and editing. **Bilal Aslam:** investigation, methodology. **Abdullah Saud:** formal analysis, writing – review and editing. **Vohra Maham Hassan:** data curation, writing – review and editing. **Aminullah Betanai:** data curation, formal analysis, investigation. **Muhammad Muneeb:** investigation, methodology, project administration. **Said Hamid Sadat:** supervision.

## Funding

The authors have nothing to report.

## Consent

Written informed consent was obtained from the patient to publish this report in accordance with the journal's patient consent policy.

## Conflicts of Interest

The authors declare no conflicts of interest.

## Data Availability

The data that support the findings of this study are available on request from the corresponding author. The data are not publicly available due to privacy or ethical restrictions.
